# Quantitative Metabolomics by ^1^H-NMR and LC-MS/MS Confirms Altered Metabolic Pathways in Diabetes

**DOI:** 10.1371/journal.pone.0010538

**Published:** 2010-05-10

**Authors:** Ian R. Lanza, Shucha Zhang, Lawrence E. Ward, Helen Karakelides, Daniel Raftery, K. Sreekumaran Nair

**Affiliations:** 1 Endocrinology Research Unit, Division of Endocrinology, Mayo Clinic College of Medicine, Rochester, Minnesota, United States of America; 2 Department of Chemistry, Purdue University, West Lafayette, Indiana, United States of America; Hong Kong University, Hong Kong

## Abstract

Insulin is as a major postprandial hormone with profound effects on carbohydrate, fat, and protein metabolism. In the absence of exogenous insulin, patients with type 1 diabetes exhibit a variety of metabolic abnormalities including hyperglycemia, glycosurea, accelerated ketogenesis, and muscle wasting due to increased proteolysis. We analyzed plasma from type 1 diabetic (T1D) humans during insulin treatment (I+) and acute insulin deprivation (I-) and non-diabetic participants (ND) by ^1^H nuclear magnetic resonance spectroscopy and liquid chromatography-tandem mass spectrometry. The aim was to determine if this combination of analytical methods could provide information on metabolic pathways known to be altered by insulin deficiency. Multivariate statistics differentiated proton spectra from I- and I+ based on several derived plasma metabolites that were elevated during insulin deprivation (lactate, acetate, allantoin, ketones). Mass spectrometry revealed significant perturbations in levels of plasma amino acids and amino acid metabolites during insulin deprivation. Further analysis of metabolite levels measured by the two analytical techniques indicates several known metabolic pathways that are perturbed in T1D (I-) (protein synthesis and breakdown, gluconeogenesis, ketogenesis, amino acid oxidation, mitochondrial bioenergetics, and oxidative stress). This work demonstrates the promise of combining multiple analytical methods with advanced statistical methods in quantitative metabolomics research, which we have applied to the clinical situation of acute insulin deprivation in T1D to reflect the numerous metabolic pathways known to be affected by insulin deficiency.

## Introduction

Insulin is a pivotal hormone regulating the metabolism of key macronutrients such as carbohydrate, fat, and protein to maintain metabolic homeostasis. Insulin deficiency induces a variety of metabolic derangements in type 1 diabetes. Elevated plasma glucose, ketones, free fatty acids, and branched-chain amino acids (BCAA) are hallmarks of untreated type 1 diabetes [1], [2], [3]. Underlying metabolic pathways that contribute to these effects include increased hepatic gluconeogenesis [4], increased glycogenolysis [5], increased flux through the glucose-alanine and Cori cycles [6], [7], increased protein breakdown in skeletal muscle [8], [9], [10], [11], and increased splanchnic protein synthesis [9]. Insulin treatment ameliorates the catabolic state in type 1 diabetic people [9], [12].

We recently studied C-peptide-negative type 1 diabetic subjects while treated with insulin and again during acute insulin deprivation (8 hours) [13]. We found that insulin deficiency altered skeletal muscle bioenergetics, as evidenced by significantly reduced skeletal muscle mitochondrial ATP production capacity and decreased transcript levels of several genes involved in mitochondrial function [13]. This study recapitulated a new role for insulin as a regulator of skeletal muscle mitochondrial function. In another report from the same cohort of patients, we report that insulin treatment and deprivation induced significant changes in the synthesis rates of individual plasma proteins, which are mostly synthesized in the liver [14]. In the present study, we measured the concentrations of plasma biomolecules in this well-characterized cohort using proton magnetic resonance spectroscopy (^1^H-MRS) and liquid chromatography tandem mass spectrometry (LC-MS/MS). Our objective was to illustrate the utility of a combination of analytical methods and multivariate statistical analysis for detecting a metabolic fingerprint that reflects known pathways that are altered with disease. Herein we apply this to the clinical situation of acute insulin deprivation in a cohort of type 1 diabetic patients in whom we have previously characterized several altered metabolic pathways [13], [14].

## Results

### Contrasting insulin treatment and deprivation in type 1 diabetes

We obtained plasma samples from 7 c-peptide negative type 1 diabetic individuals (T1D) and 7 non-diabetic controls (Con) that were matched for age (T1D = 31.1±2.9 yrs, Con = 30.2±3.4 yrs), body mass (T1D = 80.2±4.7kg, Con = 81.9±7.4 kg) and BMI (T1D = 26.5±1.2 kg/m^2^, Con = 25.2±1.3 kg/m^2^). Type 1 diabetic people were studied while treated with insulin and also after 8 hours of insulin deprivation. We performed metabolic profiling of plasma samples using a combination of ^1^H-NMR and an LC-MS/MS based method to measure plasma amino acid metabolite concentrations. We observed the expected elevations in plasma glucose (∼4 fold), beta-hydroxybutyrate (∼5 fold), acetone (∼9 fold), and acetoacetate (∼40 fold) in insulin deprived individuals compared to the insulin treated state and non-diabetic controls (Table 1). We previously reported that urinary nitrogen excretion rate increased over 8 hours of insulin deprivation in these individuals, however at the 8 hour time point, ammonia levels in plasma and urine were 16% and 70% lower, respectively, in insulin deprived compared to insulin treated and control subjects (Table 2). Plasma 3-methylhistidine levels, a marker of skeletal muscle protein degradation, were similar across all groups (Table 2).

**Table 1:** Micromolar concentrations of plasma metabolites measured by ^1^H magnetic resonance spectroscopy.

	T1D	T1D	Control	P	P	P
	(insulin treated)	(insulin deprived)	(non-diabetic)	treated v. deprived	treated v. control	control v. deprived
Glucose (mM)	6.4±1.3	22.5±2.0	5.7±1.1	<0.0001	0.809	<0.0001
Allantoin	4.0±3.4	9.4±1.0	4.6±2.7	0.017	0.809	0.014
Valine	160.5±37.8	242.8±51.3	172.6±36.4	0.004	0.809	0.026
β-hydroxybutyrate	93.0±141.5	1392.1±834.2	68.6±128.3	0.017	0.809	0.014
Acetone	99.3±52.1	757.2±421.8	75.7±46.5	0.017	0.809	0.014
Acetoacetate	26.3±14.8	205.4±126.5	23.5±17.4	0.021	0.809	0.021
Acetate	41.9±9.2	69.5±21.2	33.3±14.3	0.026	0.809	0.014
Formate	14.8 ±2.0	12.9±4.0	13.4±2.7	0.459	0.809	0.841
Citrate	95.7±34.9	123.8±57.4	106.3±57.3	0.459	0.809	0.737
Alanine	277.1±115.6	233.4±48.1	264.9±60.0	0.535	0.809	0.468
Tyrosine	33.6±11.2	32.1±6.7	27.7±10.9	0.901	0.809	0.542
Creatinine	46.3±8.9	47.9±11.5	48.7±14.7	0.901	0.809	0.915
Lactate	427.4±173.4	435.9±116.2	361.7±67.2	0.986	0.809	0.306
Histidine	83.7±7.7	83.8±14.5	88.0±19.1	0.987	0.809	0.762

Data are presented as means±SEM. P values reflect Benjamini–Hochberg correction.

**Table 2:** Micromolar concentrations of 20 standard amino acids (top panel) and amino acid metabolites (bottom panel) measured by LC-MS/MS.

	T1D	T1D	Control	P	P	P
	(insulin treated)	(insulin deprived)	(non-diabetic)	treated v. deprived	treated v. control	control v. deprived
Valine	189.4±14.5	303.5±28.2	213.5±11.8	0.003	0.616	0.078
Leucine	106.3±9.7	210.1±20.3	117.6±6.5	0.005	0.743	0.010
Isoleucine	52.2±3.5	114.1±13.0	58.5±4.1	0.005	0.653	0.010
Phenylalanine	38.1±1.1	44.1±2.1	42.4±1.4	0.031	0.403	0.759
Glutamate	113.3±20.3	88.7±18.5	108.4±30.2	0.039	0.899	0.805
Glycine	200.6±12.4	150.0±20.7	193.2±21	0.062	0.862	0.516
Tyrosine	46.0±3.6	52.0±1.7	45.0±3.3	0.064	0.899	0.417
Cystine	13.5±6.8	13.0±6.8	21.4±6.4	0.201	0.747	0.678
Threonine	95.2±4.1	87.4±9.6	121.3±12.1	0.659	0.462	0.351
Glutamine	337.9±32.9	310.5±36.2	399.7±41.1	0.659	0.653	0.489
Serine	88.9±8.0	80.1±6.6	93.4±3.5	0.659	0.832	0.417
Histidine	62.0±1.7	63.1±4.3	68.4±8.4	0.659	0.747	0.805
Tryptophan	33.6±2.3	37.6±4.3	31.7±2.4	0.659	0.832	0.603
Asparagine	36.0±4.0	34.0±5.5	43.1±1.8	0.659	0.468	0.417
Alanine	229.4±22.6	212.7±22.5	219.5±15.5	0.759	0.849	0.955
Arginine	67.2±6.8	72.9±9.0	82.4±6.0	0.775	0.468	0.678
Lysine	147.5±7.0	149.8±11.8	151.5±5.5	0.971	0.849	0.979
Proline	145.9±9.3	147.9±9.5	140.7±9.9	0.971	0.849	0.805
Methionine	14.0±1.1	13.8±2.0	16.2±0.8	0.971	0.468	0.603
Aspartate	6.5±0.9	6.4±1.3	5.4±1.0	0.971	0.747	0.759
α-aminoadipic acid	0.7±0.1	1.3±0.2	0.9±0.1	0.020	0.403	0.273
B-aminoisobutyate	1.4±0.2	2.0±0.2	2.5±0.4	0.033	0.403	0.603
Ornithine	43.5±3.1	52.6±4.3	47.7±3.6	0.049	0.747	0.678
α-amino-N-butyrate	23.6±1.6	31.9±2.1	25.7±1.2	0.052	0.736	0.156
Ammonia	121.0±18.6	100.4±17.2	109.3±28.9	0.088	0.856	0.955
Cystathionine 1	0.07±0.04	0.18±0.08	0.18±0.04	0.090	0.460	0.991
Hydroxyproline	7.9±0.5	6.4±0.7	6.5±0.4	0.224	0.460	0.979
Taurine	35.0±1.9	37.4±1.8	39.4±1.4	0.224	0.462	0.678
B-alanine	2.9±0.3	2.7±0.3	2.6±0.2	0.333	0.747	0.979
1-Methylhistidine	21.0±6.4	20.4±6.4	16.2±3.1	0.659	0.747	0.766
Allo-isoleucine	0.9±0.1	1.0±0.3	0.8±0.1	0.659	0.747	0.678
Hydroxylysine-2	0.70±0.04	0.66±0.05	0.78±0.04	0.659	0.616	0.417
Hydroxylysine-1	0.34±0.02	0.32±0.03	0.33±0.02	0.659	0.849	0.979
3-Methylhistidine	4.8±0.6	4.7±0.6	4.7±0.4	0.659	0.899	0.991
Citrulline	26.9±2.2	25.9±1.8	28.4±2.0	0.795	0.843	0.678
Ethanolamine	6.4±0.4	6.4±0.3	7.5±1.4	0.795	0.747	0.759
Sarcosine	1.1±0.1	1.0±0.2	1.1±0.2	0.759	0.899	0.910
γ-amino-N-butyrate	0.12±0.01	0.11±0.01	0.13±0.01	0.947	0.616	0.524
Phosphoethanolamine	0.29±0.16	0.29±0.20	0.61±0.12	0.957	0.489	0.516

Data are presented as means±SEM. P values reflect Benjamini–Hochberg correction.

### Evidence of altered protein metabolism revealed by LC-MS/MS analyses of plasma

We profiled physiological amino acids and amino acid metabolites in plasma samples using a LC-MS/MS method with Waters MassTrack Amino Acid Analysis Solution (Waters, Milford, MA). We monitored the concentrations of 44 metabolites, of which 41 were reliably detected in all samples, including all 20 standard amino acids (Supplemental material, Figure S1). Absolute quantitation was performed using a set of amino acid standards including glutamine, tryptophan, allo-isoleucine, and norvaline. The reproducibility of the LC-MS/MS approach for measuring amino acids and amino acid metabolites was found to be high (average CV = 9.5%, see Table S1, supplemental materials) based on repeated measurements over a 1-month period using a plasma quality control sample (N = 19 replicates). We found that insulin deprivation significantly (P<0.05) increased the levels of 5 amino acids (leucine ∼2 fold, isoleucine ∼2 fold, valine ∼1.6 fold, phenylalanine ∼1.2 fold, tyrosine ∼1.1 fold, Table 2) and significantly decreased the levels of 3 amino acids (glycine ∼1.3 fold, glutamate ∼1.3 fold, threonine ∼1.1 fold, Table 2), while the remaining 12 amino acids were unchanged compared to the same individuals treated with exogenous insulin and non diabetic controls (Table 2). Twenty-one additional amino acid metabolites were detected and quantified, of which 5 significantly increased and 1 significantly decreased with insulin deprivation (Table 2).

### Spectral profile of treated and uncontrolled type 1 diabetes

Plasma samples were also analyzed by ^1^H nuclear magnetic resonance spectroscopy. Fourteen plasma metabolites were identified based on their characteristic chemical shifts and multiplicities [15] (Supplemental material, Figure S2) and quantified by integrating peak areas relative to a reference signal from 3-(Trimethylsilyl)propionic acid-d_4_ sodium salt (TSP). These NMR analyses revealed the expected elevations in plasma glucose and ketone levels with insulin deprivation (Table 1). Although the majority of the measured metabolites were unique to each analytical technique, there were 4 amino acid metabolites that were measured in common with LC-MS/MS and ^1^H-NMR (valine, alanine, tyrosine, histidine, tables 1 and 2). Both methods revealed similar trends when comparing insulin treated and deprived type 1 diabetic people and non-diabetic controls. In agreement with the LC-MS/MS data, NMR analyses indicated elevated branched-chain amino acid (valine ∼1.6 fold) concentrations with insulin deprivation, but no change in several other amino acids (alanine, tyrosine, histidine) (Table 1).

Several metabolites involved in cellular energy metabolism were measured by ^1^H-MRS (e.g., acetate, formate, citrate, creatinine, lactate). Of these metabolites, only acetate exhibited elevated levels in insulin-deprived type 1 diabetic individuals (Table 1). Allantoin, a product of nonenzymatic urate oxidation, was significantly elevated with insulin deprivation, indicative of increased oxidative stress when insulin is withdrawn from patients with type 1 diabetes (Table 1).

### Multivariate statistical analysis differentiates plasma based on metabolites other than glucose

**Figure 1:**
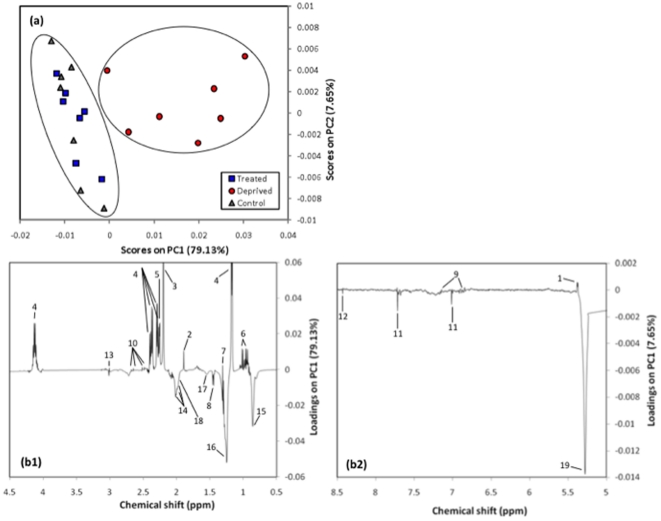
Scores plot (a) and PC1 loadings plot (b1 and b2) from the PCA of ^1^H NMR spectra of plasma from insulin deprived patients, treated patients and healthy people (glucose and residual water peaks were removed prior to PCA). Intensities of the NMR variables were normalized using the total sum of intensities. The data were analyzed after being mean-center scaled. Identified metabolites: 1, allantoin; 2, acetate; 3, acetone; 4, 3-hydroxybutyrate ; 5, acetoacetate; 6, valine; 7, lactate; 8, alanine; 9, tyrosine; 10, citrate; 11, histidine; 12, formate; 13, creatinine; 14, glycoprotein N-acetyl groups; 15, lipid:CH_3_; 16, lipid:CH_2_; 17, lipid:CH_2_CH_2_-CO;18, lipid:CH_2_-C = C;19, Lipid:fatty acyl groups = CH.

The NMR data were further analyzed by principal component analysis (PCA) using the full, processed ^1^H NMR spectra with glucose regions removed to differentiate the plasma of insulin-deprived type 1 diabetic people from insulin-treated T1D and non-diabetic controls. The PCA score plots of PC1 versus PC2 revealed that data points, each representing the ^1^H spectrum of a single participant, were clustered in a way that allowed insulin-deprived T1D to be clearly differentiated from insulin-treated T1D and controls along principal component 1 (PC1, Figure 1a). The loading plots corresponding to the aliphatic region (Figure 1b1) and aromatic region (Figure 1b2) indicate that ketones, lactate, and allantoin strongly contribute to the separation of NMR spectra from the different groups along PC1. The PCA analyses were performed after removing the glucose regions to allow differentiation of spectra without the overriding influence of the strong glucose signals.

### The integration of NMR and MS data using correlation matrices

**Figure 2:**
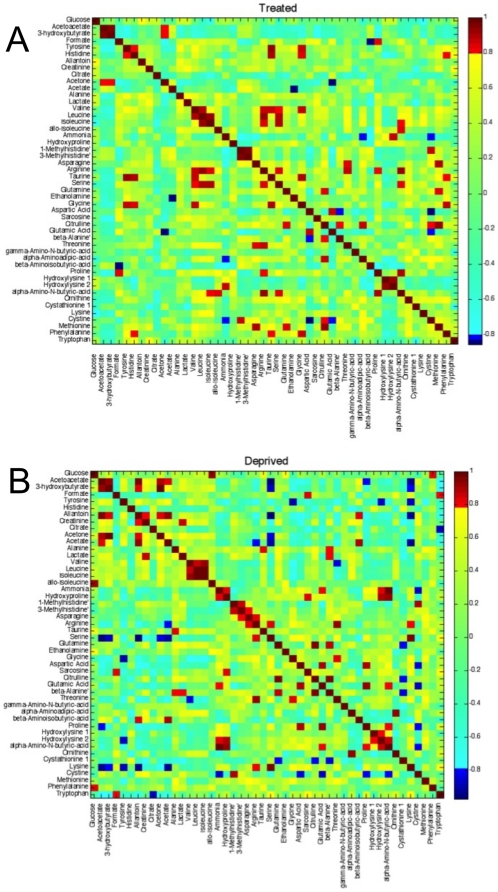
Pearson's correlations of the quantities of the 48 plasma metabolites measured by LC-MS/MS and ^1^H-NMR in type 1 diabetics who were insulin treated (a) or insulin deprived (b).

**Figure 3:**
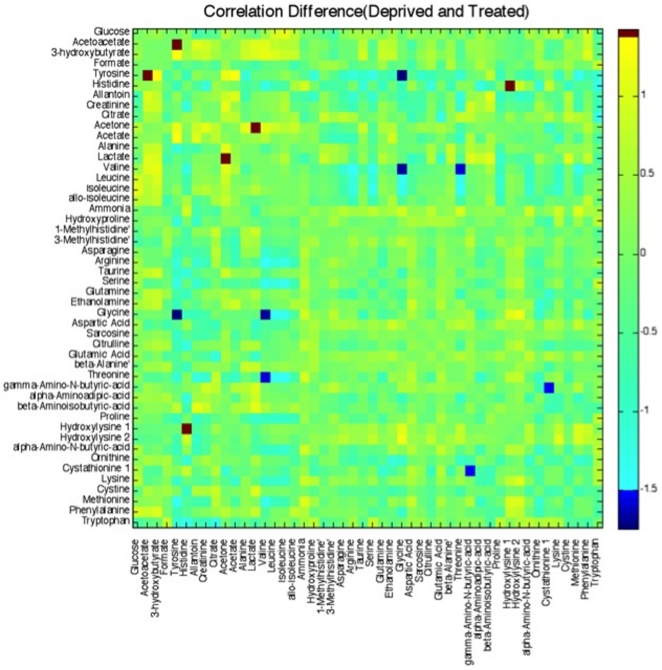
The difference in correlation of the quantities of the 48 metabolites measured by LC-MS/MS and ^1^H-NMR between type 1 diabetics during insulin deprivation and insulin treatment.

We generated correlation matrices for the plasma metabolites measured by both MS and NMR to create a compendium metabolic profile that integrates the complementary information from the two analytical methods. For metabolites measured in common by both NMR and MS, correlation matrices included data measured by NMR. Separate correlation matrices were created for insulin treated T1D (Figure 2a) and insulin deprived T1D (Figure 2b). The correlation plots reveal a wide range of correlation coefficients among all of the measured metabolites, ranging from 1.0 (maximum positive correlation) to −1.0 (maximum anticorrelation) and 0 indicating no correlation. Figures 2a and 2b illustrate that several high positive (red, yellow regions) or negative (blue regions) correlations were observed among several metabolites during conditions of insulin treatment and deprivation in the same T1D individuals. It is of interest to determine which correlations between specific metabolites were significantly altered by the presence or absence of insulin. This information can be gleaned from a side-by-side comparison of the two correlation plots (Figures 2a and 2b) or, more easily, from the correlation difference matrix generated in Figure 3. Pixels colored red or yellow indicate that the correlation coefficient between two metabolites became less positive or more negative with insulin treatment compared to insulin deprivation. Conversely, dark blue pixels represent a shift toward more positive correlation between two metabolites with insulin treatment compared to insulin deprivation. From the correlation difference matrix, it is apparent that the ketone levels (acetoacetate, 3-hydroxybutyrate, acetone) were positively correlated with plasma lactate during insulin deprivation, but negatively correlated with lactate during insulin treatment. Similarly, ketones were also positively correlated with several amino acids such as tyrosine, valine, leucine, and phenylalanine during insulin deprivation, but this correlation became negative with insulin treatment. Citrate, another metabolite related to cellular energetics, was negatively correlated with glutamate during insulin deprivation but the relationship between these two metabolites became positive with insulin treatment. During insulin deprivation, elevated allantoin was positively correlated with ketones, acetate, creatinine, and lysine, but negatively correlated with serine, arginine, glycine, and aspartate. These relationships were largely reciprocal with insulin treatment.

## Discussion

Technological advances within the past 10 years have fueled the rapid emergence of the field of metabolomics [15]. Analytical methods such as nuclear magnetic resonance spectroscopy and mass spectrometry are capable of measuring the concentrations of a large number of metabolites from body fluids, allowing potential disease biomarkers and altered biochemical pathways to be identified [16], [17], [18], [19]. Metabolomics has recently provided insights into altered metabolic pathways in diabetes induced by streptozotocin exposure in rats [6]. Specific metabolic derangements linked, in part, to altered gut microbial metabolism were revealed through the use of correlation matrices and multivariate statistics. In humans, metabolomics has recently been used to link serum metabolite profiles to clinical phenotypes and complications of type I diabetes [20] and to identify early biomarkers to predict the onset of the disease [21], [22]. While it is currently known that insulin deficiency in T1D has profound effects on many metabolic pathways, the use of large scale metabolic profiling in revealing these altered networks has not been fully explored in humans. In this study, we analyzed plasma from type 1 diabetic humans in an insulin-treated state and after withdrawing insulin for 8 hours and non-diabetic controls. We quantified several plasma metabolites using ^1^H NMR and amino acid metabolites by LC-MS/MS. This complementary analytical information was paired with multivariate statistical methods to highlight several metabolic networks known to be altered in untreated type 1 diabetes.

The ^1^H-NMR analyses revealed elevated plasma glucose, ketones, and branched-chain amino acids (BCAA), which are well-known hallmarks of insulin deficiency in type 1 diabetes [2], [3]. We anticipated that lactate and alanine would be elevated in the plasma of insulin-deprived individuals, consistent with increased gluconeogenesis through the glucose-alanine and Cori cycles as previously shown in diabetic rats [6] and humans [7]. In contrast, we found that plasma alanine was similar in insulin deprived compared to treated T1D. We also found modest, non-significant increases in plasma lactate with insulin deprivation. Notwithstanding, principal component analysis revealed that lactate was a strong contributor to differentiation of plasma from insulin treated and insulin deprived type 1 diabetic individuals. These data preclude any definitive conclusions concerning the involvement of the Cori cycle or glucose-alanine cycle.

We find that branched chain amino acids are elevated in plasma of untreated T1D, consistent with previous reports [1], [2]. Elevated BCAA are generally attributed to increased muscle protein breakdown and release of amino acids from muscle proteins [10], [23], [24] and liver [9]. We were surprised to find that after 8 hours of insulin deprivation, plasma ammonia levels (measured by LC-MS/MS) showed a trend (p = 0.088) toward lower levels in insulin-deprived vs. treated T1D, even though these patients exhibited greater urinary nitrogen loss over the first 6 hours of insulin deprivation [13]. There are several probable explanations for this finding. First, it is likely that transamination-deamination and reamination occur during acute insulin deficiency and some nitrogen is spared by synthesis of gluconeogenic precursors (e.g., alanine, glutamine) from amino acid precursors such as alpha-ketoglutarate. Our data do not support this possibility, since neither glutamine or alanine levels differed with insulin deprivation or treatment. Second, it is possible that increased urea cycle flux may buffer the appearance of ammonia in the blood by detoxifying ammonia that would otherwise accumulate as a by-product of amino acid catabolism. Citrulline and ornithine are non-proteinogenic amino acids that are involved in the urea cycle. Although citrulline levels did not differ between I+ and I−, ornithine levels were higher in I− vs. I+, making it difficult to ignore the possibility that increased urea cycle flux may temper the increase in plasma ammonia levels during acute insulin deprivation.

We found that plasma 3-methylhistidine (3-MH) levels were similar in insulin treated and deprived T1D, suggesting that myofibrillar protein degradation is not likely to be affected by short-term insulin deprivation. The urinary excretion of 3-MH is commonly used as an index of the rate of muscle proteolysis when subjects are eating a meat-free diet [25]. Although the subjects in the present study were not on a meat-free diet, they were given a weight-maintaining diet (carbohydrate∶protein∶fat = 55∶15∶30%) for three days prior to the study and remained in the fasting state after a standard meal at 16:00 hours on evening prior to the study day. This dietary control would minimize any dietary differences across the conditions that could confound the interpretation of 3-MH levels as a marker of muscle protein breakdown. One potential caveat is that our interpretation of 3-MH is based on net concentrations measured in the plasma, which provides no information regarding its appearance and disappearance. However, since 3-MH is not reutilized *in vivo* and renal function is normal, its excretion in urine reflects myofibrillar degradation with minimal contribution from gut. The absence of any increase in plasma 3-MH with insulin deprivation suggests that myofibrillar protein degradation is not likely affected by short-term insulin deprivation. Thus, the robust increase in muscle protein degradation that has been documented during short-term insulin deprivation [9] may represent non-myofibrillar muscle proteins.

In spite of a fairly comprehensive understanding of the effects of diabetes on carbohydrate metabolism, much less is known about the impact of this disease on protein metabolism. In this study, we used LC-MS/MS to measure the abundance of plasma amino acids and amino acid metabolites, which reflect the balance between AA uptake and release by various tissues that are influenced by changes in protein metabolism in response to the presence or absence of insulin and ketone levels. Numerous simultaneous processes will influence the plasma amino acid profile during insulin deprivation and insulin treatment. First, insulin acutely regulates gluconeogenesis by direct effects on amino acid balance across the splanchnic bed [26]. Second, insulin regulates protein synthesis and breakdown [8], [9], [10], [11]. We recently measured the individual synthesis rates of 41 plasma proteins during insulin treatment and deprivation in T1D in this cohort of participants [14]. This study demonstrated that the synthesis rates of some plasma proteins were increased with insulin deprivation while others decreased or did not change. Other studies indicate that some amino acids are released by skeletal muscle and taken up by the splanchnic region in response to insulin deprivation [9]. Third, plasma amino acid levels are also influenced by peripheral oxidation, particularly BCAA, which are mainly oxidized in skeletal muscle [4]. Indeed, the plasma levels of BCAA are often proposed as indices of metabolic control in type 1 and type 2 diabetes [2], [27]. Furthermore, high ketone levels also inhibit hepatic gluconeogenesis, stimulate splanchnic protein synthesis, and may or may not inhibit muscle protein degradation [28], [29]. Taken together, there are many concurrent physiological processes that are influenced by the presence or absence of insulin that can impact plasma amino acid levels. In this study, we used a relatively straightforward LC-MS/MS method to generate a profile of plasma amino acids that is characteristic of the net effect of many processes that are altered during insulin deprivation.

Parallel measurements by ^1^H-NMR revealed several changes in plasma metabolites that are distinct and complementary to the plasma amino acid metabolite profile measured by LC-MS/MS (Table 1). Using a quantitative metabolomics approach, we were able to use the ^1^H-NMR data to differentiate plasma from T1D who were either insulin treated or insulin deprived (Figure 1). Spectra from insulin treated vs. insulin deprived T1D were differentiated by PCA based largely on elevated ketones, lactate, and allantoin levels in deprived patients. Acetate levels were also significantly elevated in insulin-deprived T1D patients, although did not contribute substantially to PCA loading plots. Compilation of information from PCA, correlation matrices, and individual metabolite levels reveal a profile of altered bioenergetics, which is consistent with impaired mitochondrial function in the absence of insulin that we have previously reported in these individuals [13]. Original observations in this cohort of people indicate that insulin deprivation decreases mitochondrial ATP production [13]. Furthermore, gene array studies showed that insulin deprivation also decreased the transcript levels of genes involved in oxidative phosphorylation, which was validated by RT-PCR-based measurements of cytochrome c oxidase and ATP synthase, and mitochondrial transcription factor A [13]. Impairments at one or more levels of oxidative phosphorylation would be expected to substantially alter the redox state of the mitochondria, effectively slowing the entry of acetyl CoA into the tricarboxylic acid cycle and potentially leading to accumulation of cytosolic acetyl CoA. This effect is compounded by the conversion of citrate to acetyl CoA by the enzyme ATP citrate lyase [30]; an effect that is likely to contribute to the NMR-observable increases in plasma acetate. Plasma lactate levels were not significantly different in I+ and I−, but PCA revealed lactate as an important factor that contributed to the metabolic differentiation of I+ and I−. Although lactate is an important mitochondrial substrate and gluconeogenic precursor [31], its accumulation in plasma is a reflection of an imbalance between energy demand and supply by the mitochondria. There are several potential factors that are likely to explain elevated plasma lactate in response to insulin deprivation. First, lactate may accumulate as a result of decreased entry of acetyl CoA into the TCA cycle due to impaired mitochondrial ATP synthesis, which was demonstrated in these individuals [13]. Second, the conversion of pyruvate to acetyl CoA is likely decreased under these conditions due to the absence of insulin's effect on activating pyruvate dehydrogenase. Others have demonstrated increased urine lactate levels and TCA cycle disturbances in rats treated with streptozotocin [6].

As previously reported in a rat model of T1D [6], we observed increased plasma acetate during acute insulin deprivation. Several factors may contribute to rising acetate levels with insulin deprivation, including 1) impaired mitochondrial substrate oxidation, 2) induction of acetyl-CoA synthetase 2 under ketogenic conditions [32], 3) increased acetate release from the liver [32], and 4) a defective acetate switch and exogenous acetate production by gut microbes [6]. We did not observe significant correlations among acetate, lactate, or other metabolites (ethanol, succinate, formate) that would indicate a bacterial origin of acetate [33]. Notwithstanding, the notion of microbial origin of many plasma metabolites that are differentially elevated in controlled vs. uncontrolled diabetes should not be excluded and awaits further investigation.

Increased oxidative stress is a well-documented hallmark of diabetes and hyperglycemia [6], [33], [34]. The elevated plasma allantoin in insulin deprived T1D in the present study is consistent with this notion, as allantoin reflects non-enzymatic uric acid oxidation and is used as a biomarker of oxidative stress in humans [35]. Oxidative stress during insulin deprivation appears to be due, in part, to activation of NADPH oxidase and uncoupling of endothelium nitric oxide synthase [36]. However, it is also possible that mitochondrial dysfunction may be a determinant of increased oxidative stress during acute insulin deprivation. Mitochondrial superoxide formation is largely dependent on the mitochondrial membrane potential (Δψ), which would be expected to be elevated when ATP synthesis is impaired in the presence of excess substrate. In support of this notion, others have found that hyperglycemia increases superoxide production in endothelial cells by oversupply of respiratory chain reducing equivalents and elevated Δψ [37]. In sum, allantoin represents a convenient biomarker of oxidative stress due to its appearance in the ^1^H-NMR spectrum of human plasma and illustrates how insulin deprivation provokes oxidative stress, possibly by increasing the rate of superoxide production as a consequence of decreased mitochondrial phosphorylation activity.

By coupling the high sensitivity and specificity of MS with highly quantitative and reproducible nature of NMR spectroscopy, it is possible to perform more comprehensive metabolic profiling than allowed by each analytical method alone. In the current study, we integrated information from the two distinct analytical methods with multivariate statistical analyses to generate a plasma metabolite profile that is characteristic of several underlying physiological processes that are known to be altered by short-term insulin deprivation in type 1 diabetic people (e.g., mitochondrial dysfunction, oxidative stress, protein synthesis, degradation, and oxidation, gluconeogenesis, and ketogenesis). The ability to probe multiple unique plasma metabolites simultaneously with complementary analytical techniques holds much promise for revealing altered metabolic pathways with disease, assessing diabetic complications, and monitoring the efficacy of therapeutic interventions.

## Materials and Methods

### Study Population

C-peptide negative type 1 diabetic patients (n = 9) and age- and BMI-matched non-diabetic controls (n = 9) were recruited from the local community (Olmsted County, Minnesota) and provided written informed consent approved by the Mayo Clinic Institutional Review Board and in accordance with the Declaration of Helsinki. All study volunteers were screened with detailed medical history, physical exam, and hematological and biochemical profile. Potential volunteers were excluded if they exhibited renal insufficiency, heart disease, peripheral vascular disease, neuropathy, poor wound healing, or use of beta-blockers, tricyclic antidepressants, or anticoagulants.

### Study protocol

The details of this study have been published previously [13]. Type 1 diabetic subjects completed two studies; one during insulin treatment and one during insulin deprivation with 1–2 weeks between study days. For three days prior to each study, subjects consumed a weight-maintaining diet (energy content as carbohydrate∶protein∶fat = 55∶15∶30%) prepared by the Mayo CTSA Clinical Research Unit (CRU). During the 3 days prior to the study, subjects who were taking multiple daily insulin injections were instructed to use ultra rapid-acting insulin before meals and bedtime based on blood glucose concentrations. Those subjects taking long-acting insulin were instructed to discontinue dosage for 3 days prior to the study day. Patients who used insulin pumps were instructed to continue using ultra rapid-acting insulin until admission to the CRU. Subjects were admitted to the CRU at 17:00 h on the evening before each study day. A standard dinner was given at 18:00 h, after which subjects remained fasting until the completion of the study the next day. On the insulin-treated study day, human insulin was infused into a forearm vein to maintain blood glucose between 4.44 and 5.56 mmol/l overnight until 12:00 h the next day. The dose of insulin was adjusted based on plasma glucose levels every 30–60 minutes. On the insulin-deprived study day, the insulin infusion was discontinued for 8.6±0.6h. Arterialized vanous blood was obtained from a catheterized hand vein maintained at 60°C at 8 hours or insulin treatment or deprivation. Plasma was kept at −80°C until analysis.

### Mass spectrometry

Plasma samples and amino acid calibration standards were prepared with MassTrak Amino Acid Analysis Solution (AAA) kit from Waters according to instructions with slight modifications for detection on a mass spectrometer. A 10 point standard concentration curve was made from the calibration standard solution to calculate amino acid concentrations in plasma samples. A solution containing U-^13^C_4_-L-aspartic acid, U-^13^C_3_-L-alanine, U-^13^C_4_-L-threonine, U-^13^C_5_-L-proline, U-^13^C_5_-L-valine, U-^13^C_6_-leucine, U-^13^C_6_-phenylalanine all from Cambridge Isotope Laboratories, ^13^C_6_-tyrosine from Isotec, L-arginine (^15^N_2_, ^2^H_2_) from MassTrace, norvaline from Sigma dissolved in 0.01N HCl was used as the internal standard solution. Frozen plasma samples were thawed, spiked with internal standard then deproteinized with cold MeOH followed by centrifugation at 10,000 g for 5 minutes prior to derivatization according to MassTrak instructions. The amino acid derivatizing reagent used was 6-aminoquinolyl-N-hydroxysuccinimidyl carbamate. High resolution separation was done using an Acquity UPLC system, injecting 1 µl of derviatized solution, with a UPLC BEH C18 1.7 micron 2.1×150 mm column from Waters. Column flow was set to 400 µl/min with a gradient from 99.9%A to 98%B where buffer A is 1% acetonitrile in 0.1% formic acid and buffer B is 100% acetonitrile. A column temp of 43 degrees Celsius and a sample tray temp of 6% Celsius. Mass detection was completed on a TSQ Ultra Quantum from Thermo Finnigan running in ESI positive mode. A scan width of 0.002, scan time of 0.04 seconds per transition mass, collision energy of 25, collision gas pressure of 1.5 mTorr, tube lens value set to 90, monitoring a signature ion of the derivitized amines at m/z 171.04 by selected reaction monitoring. Using this method 39 amino acids and metabolites were measured as shown in Table 2 and Table S1 (supplementary data).

### ^1^H NMR spectroscopy

Frozen plasma samples were thawed and 500 µl was mixed with phosphate buffer (75 µl, 0.5M, pH 7.0), D_2_O (75 µl), 3-(Trimethylsilyl)propionic acid-d_4_ sodium salt (TSP, 5µl in D_2_O, 12.3 nmol). Any particulate matter was removed by centrifugation and 500 µl of the supernatant was transferred into 5-mm NMR tubes. All high-resolution ^1^H NMR experiments on plasma were performed at 25°C on a Bruker DRX 500 spectrometer equipped with a HCN triple resonance cryogenic probehead. ^1^H NMR spectra were acquired using the Carr-Purcell-Meiboom-Gill (CPMG) experiment with water presaturation. CPMG experiments are useful for the analysis of plasma to reduce the contribution of broad and mostly featureless protein signals. Each dataset was averaged over 32 transients using 32K time domain points. The data were Fourier transformed after multiplying by an exponential window function with a line broadening of 0.3 Hz, and the spectra were phase and baseline corrected using the Bruker XWinNMR software version 3.5. Twenty plasma metabolites were identified based on their characteristic chemical shifts and multiplicities and quantified by integrating peak areas relative to a reference signal from 3-(Trimethylsilyl)propionic acid-d_4_ sodium salt (TSP). The NMR assignments were made based on literature values, and tentative assignments made due to the overlap or slight shift in their positions were confirmed by re-recording 1H NMR spectra for before and after the addition of small quantities of the respective standard compounds.

### Statistical Analysis

Each NMR data set was binned to 4096 points (bin size = 0.003 ppm) to minimize the effects of pH and ionic concentrations. The data were aligned with reference to the TSP signal and the regions containing TSP, urea and residual water (4.6∼5.3 ppm) signals were removed. The NMR data were mean-centered and then subjected to unsupervised statistical analysis, PCA, using Pirouette software version 3.11 (Infometrix Inc., PA) to classify the samples (control or diabetic) based on the metabolites that varied the most across the entire sample set. Individual plasma metabolites determined by ^1^H NMR spectroscopy and plasma amino acids determined by UPLC-MS/MS were compared across diabetic and control subjects by unpaired t-tests and in treated vs. untreated diabetic subjects by paired t-tests. P values for individual metabolites were determined by the standard t-test. A Benjamini–Hochberg correction was applied to adjust the P values by accounting for the multiple metabolites used in the analysis. In addition, the Pearson correlations among all the metabolites (for insulin-deprive, treated and control samples separately) were calculated utilizing the R software package. The statistical significance of the correlation coefficients was tested using a t test by giving a null hypothesis (H_0_) of r = 0, where r represents the correlation coefficient. If H_0_ holds, 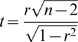 approximately follows the t distribution with degrees of freedom equal to n–2, where n represents the sample size. A low P value (<0.05) for this test means that there is evidence to reject the null hypothesis in favor of the alternative hypothesis or that there is a statistically significant relationship between the two variables (metabolites). Correspondingly, an absolute value of r larger than 0.76 is considered as a statistically significant relationship between the two metabolites.

## Supporting Information

Figure S1 Representative Chromatograms for plasma metabolites measured by LC-MS/MS. Example chromatograms of 41 analytes in plasma from a type 1 diabetic patient treated with insulin (top), deprived of insulin (middle), and a non-diabetic control subject (bottom).
(0.37 MB TIF)

Figure S2 Representative 1H-NMR spectra of plasma metabolites. Representative 1H-NMR spectra of plasma from an insulin treated type 1 diabetic patient (top), and insulin deprived type 1 diabetic patient (middle), and a non-diabetic control subject (bottom). Identified metabolites: 1, allantoin; 2, acetate; 3, acetone; 4, 3-hydroxybutyrate ; 5, acetoacetate; 6, valine; 7, lactate; 8, alanine; 9, tyrosine; 10, citrate; 11, histidine; 12, formate; 13, creatinine; 14, glycoprotein N-acetyl groups; 15, lipid:CH3; 16, lipid:CH2; 17, lipid:CH2CH2-CO;18, lipid:CH2-C = C;19, Lipid:fatty acyl groups = CH; 20: Glucose.
(1.43 MB TIF)

Table S1 Interassay variation for plasma amino acid metabolites measured by LC-MS/MS. Interassay variation of the plasma amino acid and metabolite measurements by LC-MS/MS using the Masstrak reagents. A total of 19 replicates were measured over the course of a 1 month period using a plasma quality control sample.
(0.06 MB DOC)
