# Supplementary figures and images for: Quantitative Metabolomics by 1H-NMR and LC-MS/MS Confirms Altered Metabolic Pathways in Diabetes
Source: PLoS One. 2010 May 10;5(5):e10538. doi: 10.1371/journal.pone.0010538 (PMC2866659; doi:10.1371/journal.pone.0010538)

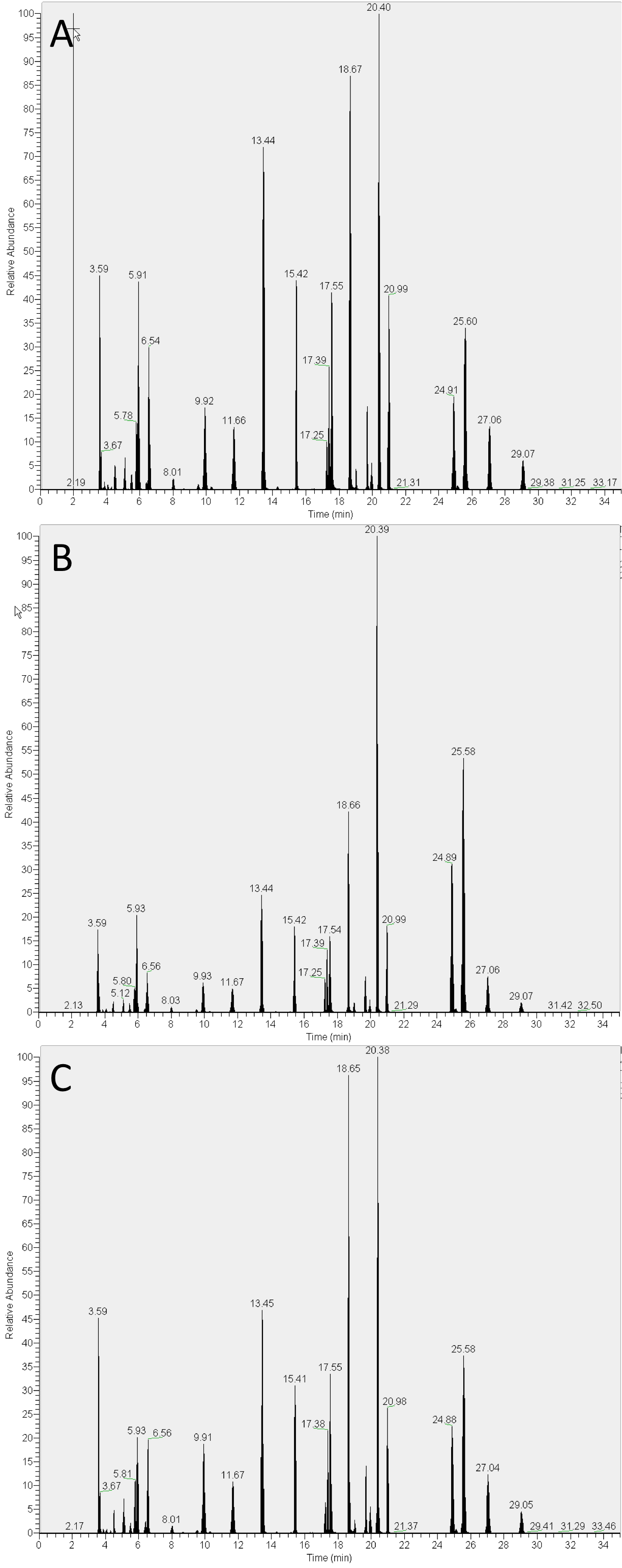

Supplement: Figure S1 — Representative Chromatograms for plasma metabolites measured by LC-MS/MS. Example chromatograms of 41 analytes in plasma from a type 1 diabetic patient treated with insulin (top), deprived of insulin (middle), and a non-diabetic control subject (bottom). (0.37 MB TIF) [file pone.0010538.s001.tif]

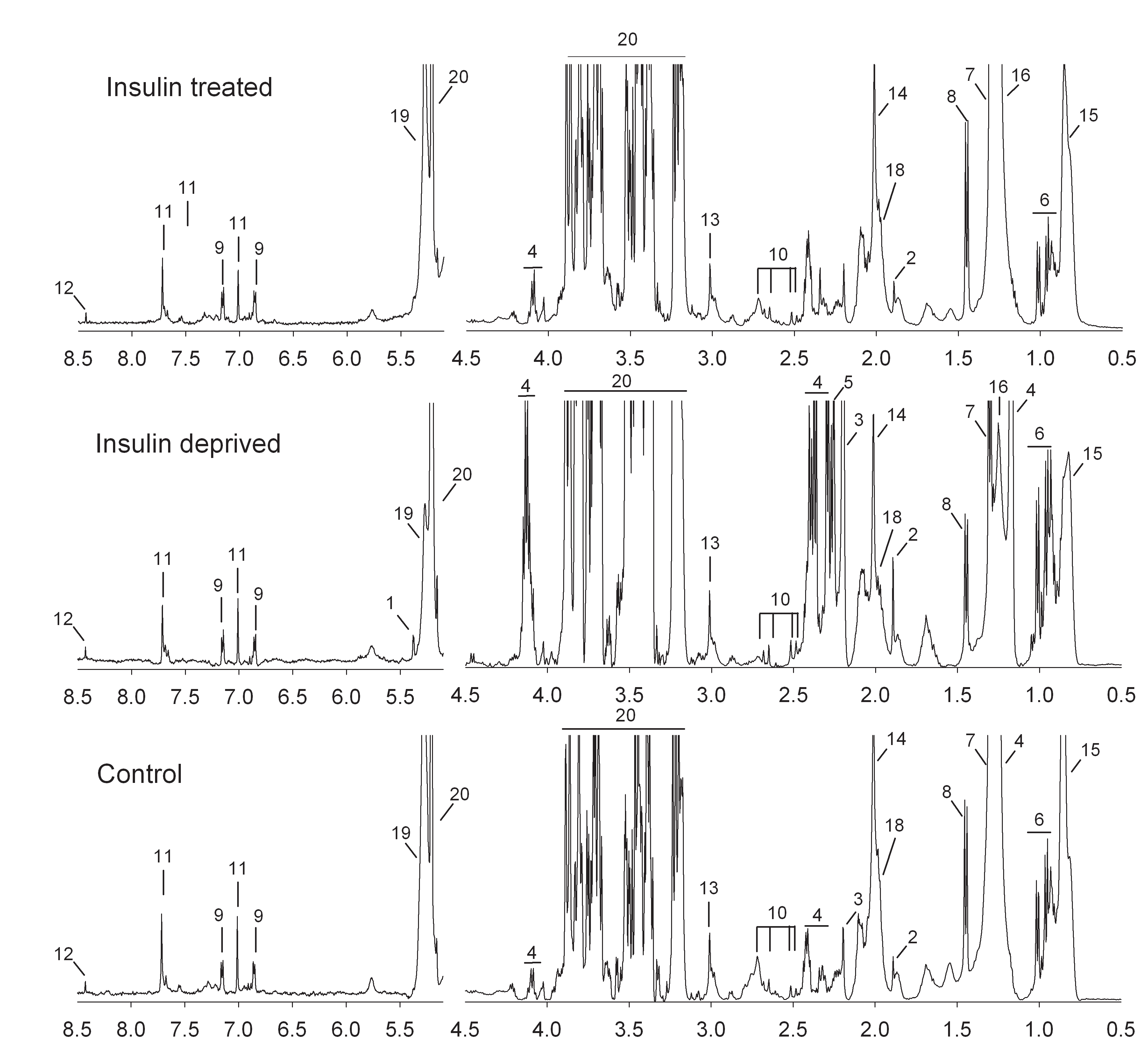

Supplement: Figure S2 — Representative 1H-NMR spectra of plasma metabolites. Representative 1H-NMR spectra of plasma from an insulin treated type 1 diabetic patient (top), and insulin deprived type 1 diabetic patient (middle), and a non-diabetic control subject (bottom). Identified metabolites: 1, allantoin; 2, acetate; 3, acetone; 4, 3-hydroxybutyrate ; 5, acetoacetate; 6, valine; 7, lactate; 8, alanine; 9, tyrosine; 10, citrate; 11, histidine; 12, formate; 13, creatinine; 14, glycoprotein N-acetyl groups; 15, lipid:CH3; 16, lipid:CH2; 17, lipid:CH2CH2-CO;18, lipid:CH2-C = C;19, Lipid:fatty acyl groups = CH; 20: Glucose. (1.43 MB TIF) [file pone.0010538.s002.tif]
