# Supplementary material for: Quantitative Metabolomics by 1H-NMR and LC-MS/MS Confirms Altered Metabolic Pathways in Diabetes
Source: PLoS One. 2010 May 10;5(5):e10538. doi: 10.1371/journal.pone.0010538 (PMC2866659; doi:10.1371/journal.pone.0010538)
Supplement: Table S1 — Interassay variation for plasma amino acid metabolites measured by LC-MS/MS. Interassay variation of the plasma amino acid and metabolite measurements by LC-MS/MS using the Masstrak reagents. A total of 19 replicates were measured over the course of a 1 month period using a plasma quality control sample. (0.06 MB DOC) [file pone.0010538.s003.doc]

Table SI: Interassay variation of the plasma amino acid and metabolite measurements by LC-MS/MS using the Masstrak reagents. A total of 19 replicates were measured over the course of a 1 month period using a plasma quality control sample.

|  | Mean (uM) | SD | CV (%) |
| --- | --- | --- | --- |
| Ammonia | 130.4 | 16.1 | 12.4 |
| Histidine | 71.6 | 5.2 | 7.2 |
| Hydroxyproline | 29.6 | 1.6 | 5.4 |
| 1-methylhistidine | 8.7 | 0.8 | 8.6 |
| 3-methylhistidine | 9.8 | 0.6 | 6.2 |
| Asparagine | 46.4 | 2.7 | 5.8 |
| Phosphoethanolamine | 3.7 | 0.4 | 12.0 |
| Arginine | 71.5 | 3.3 | 4.6 |
| Taurine | 63.1 | 3.8 | 6.0 |
| Anserine | 0.6 | 0.1 | 18.5 |
| Serine | 85.1 | 7.1 | 8.4 |
| Glutamine | 466.4 | 32.6 | 7.0 |
| Ethanolamine | 6.1 | 0.6 | 9.7 |
| Glycine | 197.6 | 13.5 | 6.9 |
| Aspartic Acid | 6.3 | 0.6 | 9.0 |
| Sarcosine | 4.7 | 0.2 | 4.1 |
| Citrulline | 36.1 | 2.7 | 7.4 |
| Glutamic Acid | 127.9 | 10.5 | 8.2 |
| Beta-alanine | 3.4 | 0.3 | 7.8 |
| Threonine | 111.4 | 3.5 | 3.2 |
| Alanine | 354.8 | 12.1 | 3.4 |
| Alpha-aminoadipic acid | 1.8 | 0.1 | 6.5 |
| Beta-aminoisobutyric acid | 0.6 | 0.1 | 11.4 |
| Proline | 178 | 4.1 | 2.3 |
| Hydroxylysine-1 | 0.7 | 0.2 | 27.6 |
| Hydroxylysine-2 | 1.6 | 0.2 | 12.1 |
| Alpha-amino-N-bytyric acid | 29.6 | 0.5 | 1.6 |
| Ornithine | 85.8 | 3.8 | 4.5 |
| Lysine | 189.5 | 11.1 | 5.9 |
| Cystine | 5.7 | 1.4 | 25.4 |
| Tyrosine | 65.6 | 3.3 | 5.0 |
| Methionine | 21.0 | 1.2 | 5.8 |
| Valine | 265.9 | 10.2 | 3.8 |
| Isoleucine | 92.7 | 7.9 | 8.6 |
| Allo-Isoleucine | 0.8 | 0.7 | 78.1 |
| Leucine | 154.6 | 4.6 | 3.0 |
| Phenylalanine | 58.3 | 2.0 | 3.5 |
| Tryptophan | 69.4 | 2.0 | 2.9 |
